# Resting-state EEG connectome topology and social norm processing in healthy young adults

**DOI:** 10.1007/s00429-026-03167-9

**Published:** 2026-07-30

**Authors:** Nicola Davide Cavallo, Simone Papallo, Marianna Chianese, Leandro Donisi, Chiara Baiano, Gabriella Santangelo, Luigi Trojano, Fabrizio Esposito

**Affiliations:** 1 Department of Psychology, University of Campania “Luigi Vanvitelli”, Caserta, Italy; 2 Department of Advanced Medical and Surgical Sciences, University of Campania “Luigi Vanvitelli”, Naples, Italy

**Keywords:** Resting-state EEG, Functional connectivity, Graph theory, Network topology, Social cognition, Social norms

## Abstract

Resting-state network topology may provide a powerful framework for understanding how large-scale brain organisation supports cognitive functions. However, the topological correlates of social cognition remain poorly characterised. This study examined whether individual differences in social cognition are associated with the global topology of the resting-state functional connectome derived from high-density EEG across canonical frequency bands. Twenty-eight healthy young adults underwent a comprehensive neuropsychological assessment, including measures of global cognition, theory of mind, empathy, and social norm processing. High-density eyes-closed resting EEG was acquired, and source-reconstructed connectivity matrices were estimated using the phase-locking value. For each frequency band, global graph-theoretical measures of segregation (average clustering coefficient, CC), integration (characteristic path length, CPL), and small-worldness were computed. We found selective associations between network topology and the intrapersonal component of social norm processing. Higher intrapersonal norm scores were related to lower CC and longer CPL, predominantly in the theta and alpha bands. These relationships held after controlling for demographic variables and were supported by multivariate analyses. No other social cognition measures showed equally significant associations with resting-state EEG topology. These findings suggest that individual variability in intrapersonal social norm processing may be associated with differences in the global organisation of the intrinsic functional connectome and highlight the relevance of investigating normative aspects of social cognition within a resting-state connectomics framework.

**Supplementary Information:**

The online version contains supplementary material available at https://doi.org/10.1007/s00429-026-03167-9.

## Introduction

Social cognition enables individuals to perceive, interpret, and predict others’ behaviour, thereby supporting adaptive interaction within complex social environments (Adolphs 2009; Van Overwalle 2009). It encompasses multiple, partially separable yet interacting constructs, including theory of mind (ToM), emotion recognition, empathy, and the knowledge and enforcement of social norms (Mitchell 2008; Van Overwalle 2009). Although considerable progress has been made in identifying the neural substrates of these functions, current models of the “social brain” remain largely region-centric or network-specific, focusing on activation patterns or connectivity strength within predefined functionally connected systems (Shamay-Tsoory and Aharon-Peretz 2007; Van Overwalle 2009; Cavallo et al. 2026). These approaches have provided important insights into the neural substrates of social cognition, yet comparatively less attention has been devoted to understanding how the global organisational properties of the entire connectivity pattern (i.e., the connectome) constrain and shape complex socio-cognitive behaviour.

Within the social cognition framework, social norms are culturally shared rules that guide expectations and evaluations of interpersonal behaviour. Intrapersonal norm reasoning, in particular, requires individuals to internally simulate their own behaviour within a social context, evaluate its appropriateness against internalised normative representations, and regulate behavioural tendencies accordingly (Van Overwalle 2009; Zinchenko and Arsalidou 2018). These processes involve the integration of self-referential evaluation, executive monitoring, and socio-emotional processing, functions that are thought to depend on distributed and interacting large-scale brain systems rather than on isolated focal regions (Bzdok et al. 2012; Bassett and Sporns 2017; Krendl and Betzel 2022). In fact, detecting norm violations and representing normative rules engage several brain regions, including the medial prefrontal, anterior cingulate, and insular cortices, which partially overlap with other social-brain systems (Xiang et al. 2013; Arioli and Canessa 2019). Meta-analytic evidence shows that different aspects of norm processing (i.e., representation vs. norm violation) recruit distinct brain regions, highlighting the value of fine-grained behavioural measures when linking social cognition to its neural correlates (Zinchenko and Arsalidou 2018). Collectively, these findings suggest that intrapersonal norm reasoning is unlikely to depend on isolated regions but rather relies on the coordinated interaction of heterogeneous large-scale systems.

Prior studies have primarily examined connectivity strength within specific networks (e.g., default mode or salience systems) in relation to ToM or empathy (Hughes et al. 2019; Christov-Moore et al. 2020; Krendl and Betzel 2022). While functional connectivity strength quantifies pairwise statistical associations between regional activity patterns, it does not capture the higher-order topological properties that define how interactions are organised across the whole brain. In contrast, graph theory provides a concise and interpretable mathematical framework to characterise the local-global topology of the functional connectome, quantifying the level of integration and segregation, which are thought to support efficient cognitive processing (Watts and Strogatz 1998; Bullmore and Sporns 2009; Rubinov and Sporns 2010). Within graph theory, the brain is modelled as nodes connected by weighted or binary edges, in which the nodes represent the brain regions and the edges the level of synchronisation between them (Sporns et al. 2005; Sporns 2018). The clustering coefficient (CC) measures the tendency of a node’s neighbours to be interconnected (Rubinov and Sporns 2010). Higher clustering reflects increased local segregation, indicating that information processing may occur within densely interconnected node groups. In contrast, characteristic path length (CPL) quantifies the average shortest topological distance between pairs of nodes, indexing the level of global integration and the potential for integrative communication across distant regions (Latora and Marchiori 2001; Rubinov and Sporns 2010). In addition, the small-world index (SW) quantifies the coexistence of high local clustering and short path length relative to comparable random networks, thereby capturing the balance between segregation and integration that characterises many complex systems, including the human brain (Bullmore and Sporns 2009; Rubinov and Sporns 2010).

From a network neuroscience perspective, cognitive functions emerge not only from local-global interactions but also from the balance between the local specialisation and distributed integration of the functional connectome that shapes how information is exchanged across subsystems (Park and Friston 2013; Bertolero et al. 2015; Bassett and Sporns 2017).

Previous studies have explored the relationship between brain topology and individual differences in cognitive performance (Park and Friston 2013; Bassett and Sporns 2017). In several domains, such as intelligence and working memory, connectome configurations characterised by shorter path length and higher clustering have often been associated with better performance (Van Den Heuvel et al. 2009; Langer et al. 2012; Cohen and D’Esposito 2016), suggesting that specific balances of integration and segregation may support effective cognitive processing (Sporns 2013; Park and Friston 2013; Bassett and Sporns 2017). However, accumulating evidence indicates that this relationship is not monotonic.

Task-based studies demonstrate that large-scale networks dynamically reconfigure between more integrated and more segregated states depending on cognitive demands (Cole et al. 2013; Shine et al. 2016). Some empirical studies suggest that higher global network efficiency may relate to slower cognitive performance (Vriend et al. 2020), and resting-state EEG evidence indicates that longer CPL may be positively associated with cognitive performance (Zakharov et al. 2020), suggesting that optimal topological configurations may be domain-sensitive.

Intrapersonal norm reasoning represents a theoretically informative test case within this framework. Unlike relatively circumscribed operations such as working memory, intrapersonal normative evaluation requires the parallel coordination of heterogeneous computational subsystems: self-referential simulation, affective appraisal, rule-based knowledge activation, and executive monitoring (Van Overwalle 2009; Bzdok et al. 2012; Zinchenko and Arsalidou 2018). Under this perspective, cognitive operations that rely on the coordinated interaction of multiple functional subsystems may not be optimally supported by global configurations that maximise either local segregation or global integration alone. Instead, they may depend on a balanced and flexible organisational regime that enables cross-system communication while preserving functional differentiation. Examining global topological properties, therefore, provides a principled approach to testing whether socio-cognitive functions such as intrapersonal norm reasoning rely on distinct integration–segregation balances compared to other cognitive domains. In this context, resting-state functional connectivity offers a parsimonious window into the intrinsic large-scale organisational architecture that constrains and shapes subsequent task-evoked processing (Sporns 2013; Raichle 2015).

Prior EEG graph-theoretical studies have also demonstrated that band-specific network topology captures individual differences in cognitive performance (Barttfeld et al. 2011; Vecchio et al. 2014; Dai et al. 2017; Zakharov et al. 2020). Different oscillatory bands appear to support distinct large-scale coordination mechanisms (Buzsáki 2006; Fries 2015), suggesting that frequency-dependent network organisation may differentially relate to cognitive functions.

Although graph metrics have successfully linked network topology to cognitive performance across several domains, to the best of our knowledge, no study has systematically examined whether resting-state EEG connectome topology indexes inter-individual variability in social norm processing in healthy adults. In the present study, we aimed at testing the hypothesis that inter-individual variability in social cognition is associated with frequency-specific patterns of resting-state EEG topology. Given the integrative and multidimensional nature of intrapersonal norm reasoning, we could expect that it would relate to global properties of network organisation reflecting the balance between segregation and integration. Furthermore, considering the functional relevance of a wide range of oscillatory bands for internally oriented and control-related processes (Buzsáki 2006; Klimesch 2012; Cavanagh and Frank 2014), we hypothesised that individual social cognition performance reflects the joint topological contribution of multiple canonical EEG bands rather than frequency-specific effects.

## Materials and methods

### Participants

Healthy young adults were recruited at the University of Campania “Luigi Vanvitelli” in December 2025. Inclusion criteria were: (i) age ≥ 18 years; (ii) absence of neurological or psychiatric disorders; (iii) normal or corrected-to-normal vision; and (iv) no history of substance abuse. All participants were instructed to refrain from consuming caffeine or other substances that could affect EEG activity in the hours preceding the recording.

Thirty-five individuals meeting these criteria were initially enrolled to undergo non-simultaneous neuropsychological assessment and resting-state EEG recording. Seven participants were excluded due to excessive EEG artefacts that prevented reliable preprocessing and source reconstruction.

The final sample, therefore, comprised twenty-eight healthy young adults (16 females; mean age = 27.04 ± 5.40 years), with a mean of 16.7 ± 2.4 years of education.

The study was conducted in accordance with the Declaration of Helsinki and was approved by the Ethics Committee of the University of Campania “Luigi Vanvitelli” (approval number: 44/2025). All participants provided their written informed consent before participation.

### Neuropsychological assessment

#### Montreal Cognitive Assessment (MoCA)

Global cognitive functioning was assessed using the MoCA (Nasreddine et al. 2005; Santangelo et al. 2015), a brief neuropsychological screening tool for cognitive impairment. The MoCA comprises 12 tasks covering multiple cognitive domains, including memory, attention, executive functions, language, visuospatial abilities, abstraction, calculation, and temporal–spatial orientation. Total scores range from 0 to 30, with higher scores reflecting better cognitive performance.

#### Social cognition

The following measures were used to assess participants’ social cognition abilities.

##### Edinburgh Social Cognition Test (ESCoT)

The ESCoT (Baksh et al. 2018; Isernia et al. 2022) assesses multiple domains of social cognition, specifically affective and cognitive ToM, as well as interpersonal and intrapersonal understanding of social norms. The instrument comprises 11 silent, cartoon-style animations (one practice and 10 test trials), each lasting approximately 30 s, depicting everyday social interactions that either conform to or violate social norms. Following each animation, a static storyboard summarising the interaction is presented and remains visible until the participants’ response is complete. After each animation, participants responded verbally to five open-ended questions assessing: (1) animation comprehension (not valid for scoring); (2) cognitive ToM (ESCoT_COGN_ToM); (3) affective ToM (ESCoT_AFF_ToM); (4) interpersonal norm evaluation (ESCoT_INTER); and (5) intrapersonal norm evaluation (ESCoT_INTRA). Interpersonal norm evaluation assesses participants’ understanding of the implicit social rules underlying the interaction and whether the character’s behaviour conforms to those rules. In contrast, intrapersonal norm evaluation assesses how participants believe they themselves would behave in the same situation. Thus, while both components involve social norm understanding, the interpersonal condition focuses on evaluating another person’s behaviour, whereas the intrapersonal condition requires participants to consider their own behaviour within the social context depicted.

The responses were transcribed by the examiner to facilitate potential inter-rater comparisons, and each was scored from 0 to 3 (max score for each animation: 12), with higher scores indicating greater integration and interpretation of relevant social and contextual information. In the Italian version of ESCoT, two items (animation 2 and 5) are not recommended because they are poorly suited to the Italian socio-cultural context (Isernia et al. 2022). For this reason, the overall task score ranged from 0 to 96, and higher scores indicate better performance in social cognition.

##### Social Norms Questionnaire (SNQ)

The SNQ (Kramer et al. 2014; Isernia et al. 2022) was administered to assess participants’ knowledge and application of implicit social norms in their cultural context. The instrument consists of 22 items describing everyday behaviours, which participants judge as either socially acceptable or unacceptable. A total accuracy score (SNQ_TOT) is computed as the sum of correct responses and ranges from 0 to 22, with higher scores indicating more accurate social norm judgments. Moreover, two error-based indices are derived: the Break score (SNQ_BREAK), reflecting inappropriate behaviours judged as acceptable, and the Overadhere score (SNQ_OVER), reflecting appropriate behaviours judged as unacceptable.

Unlike the ESCoT, which assesses social norm understanding within dynamic and context-rich social interactions, the SNQ primarily evaluates knowledge and application of culturally shared social rules through explicit acceptability judgments. Therefore, although both instruments assess aspects of social norm processing, they tap partially different components of normative social cognition.

##### Questionnaire of Cognitive and Affective Empathy (QCAE)

Empathy was measured with the QCAE (Reniers et al. 2011; Di Girolamo et al. 2019), a 31-item self-report tool rated on a 4-point Likert scale. The QCAE produces separate scores for cognitive and affective empathy, as well as a global empathy score. The cognitive empathy scale includes two subcomponents: Perspective Taking (PT), assessing the tendency to adopt another person’s viewpoint, and Online Simulation (OS), measuring the ability to imagine another person’s thoughts or feelings, often in preparation for future actions. The affective empathy scale comprises three subcomponents: Emotion Contagion (EC), assessing automatic mirroring of others’ emotions; Proximal Responsivity (PrR), measuring emotional responsiveness to people in close social or emotional proximity; and Peripheral Responsivity (PeR), evaluating empathic responses to more distant or fictional contexts, such as characters in films or books. Subscale scores are summed to obtain the two main component scores (i.e., cognitive and affective empathy). For all QCAE scales, higher scores correspond to greater self-reported empathy.

### EEG acquisition and preprocessing

Resting-state EEG data were recorded using a 64-channel electrode cap connected to a LiveAmp64 portable 24-bit amplifier (Brain Products^®^, Germany). Active Ag/AgCl electrodes were positioned according to the international 10–20 system (Jasper 1958). A high-viscosity conductive gel was applied to maintain electrode impedances below 25 kΩ, ensuring an appropriate signal recording. Signals were sampled at 500 Hz with FCz serving as the online reference. Data acquisition was performed with the Brain Vision Recorder (Brain Products^®^, Germany). Recordings were conducted in a normally lit room for 10 min, while participants kept their eyes closed and remained awake. Preprocessing was carried out in Brain Vision Analyzer 2.3.0 (Brain Products^®^, Germany). Noisy or unstable electrodes were removed based on visual inspection and offline review (number of channels: 61.14 ± 2.4) (Lantz et al. 2003). Data were filtered using a bandpass 4th-order IIR Butterworth filter with a bandwidth ranging between 0.1 and 40 Hz, and a 50 Hz notch filter to remove the contribution from known technical sources of noise. Off-line re-referencing was performed to the average signal of the mastoid electrodes (TP9, TP10), which are considered approximately neutral with respect to the neural activity recorded by the other electrodes (Hagemann et al. 2001). This ensured a temporally stable zero-potential reference, overcoming the time-varying nature of the zero-boundary curve and generating a signal resulting from the potential difference between the active electrode and the reference site (Yao et al. 2019). Ocular artefacts were corrected using independent component analysis (ICA), and components related to eye blinks and movements were identified based on activity in FP2 (vertical movements) and T7/T8 (horizontal movements). An automated procedure including raw data inspection of gradient, amplitude, and variance thresholds was exploited to identify additional artefacts and therefore exclude affected signal segments (final continuous acquisition duration: 581.19 ± 80.27 s). For the subsequent preprocessing steps, the cleaned EEG data were imported into MATLAB-R2023a (The Mathworks, Inc., www.mathworks.com). Seven band-pass filters were applied to the pre-processed signal, with appropriate bandwidths according to the seven canonical EEG frequency bands, delta (2–4 Hz), theta (4–8 Hz), alpha-1 (8–10.5 Hz), alpha-2 (10.5–13 Hz), beta-1 (13–20 Hz), beta-2 (20–30 Hz), and gamma (30–40 Hz) to reconstruct the source signals separately for each frequency band. A realistic head model was generated with the boundary element method (BEM) implemented in OpenMEEG (Gramfort et al. 2010), and based on this model, the evaluation of the leadfield matrix was computed for 4452 sources. From the computed lead fields, the minimum norm estimation (MNE) algorithm was applied to the channel time-series, resulting in 4452 source time series from the cortical brain surface. All steps were implemented in the FieldTrip toolbox (Oostenveld et al. 2011). Then, the source signals were further parcellated according to the 100-Schaefer atlas (Schaefer et al. 2018) applied to the cortical surface model, yielding 1071 vertices, which were assigned to the 100 parcels. The magnitude of the source signals within each specific parcel was averaged across vertices, resulting in 100 regional time series that were z-scored in time. Finally, phase locking value (PLV) was calculated as a statistical coupling approach for determining the functional connectivity (FC) among brain regions, yielding a 100 × 100 FC matrix for each EEG-band and subject (Lachaux et al. 1999; Aydore et al. 2013). PLV was selected as the primary connectivity metric because it quantifies the consistency of phase relationships across time and is widely used in EEG connectomics (Cohen 2014; Hatlestad-Hall et al. 2021). Although PLV may be influenced by residual volume conduction and zero-lag coupling, source reconstruction can partially mitigate these effects and provides a more anatomically interpretable estimate of functional connectivity. Compared with more conservative phase-based measures such as the phase lag index and weighted phase lag index, PLV may provide greater sensitivity to phase synchronisation, albeit with reduced specificity for non-zero-lag interactions (Stam et al. 2007; Vinck et al. 2011).

### Extraction of network graph theory metrics

Adopting the model of the brain as a connected graph (Sporns et al. 2005), we extracted the graph-theoretical metrics from the undirected weighted brain network derived for each EEG band. A proportional thresholding procedure (Jalili 2016) was commonly applied to control the different levels of density sparsity of the connectivity matrices. Herein, we adopted a cost-integration method, which includes a range of thresholds for each of which the graph-theoretical metrics of interest are extracted, and calculates the area under the curve (AUC), thereby defining an average value as a threshold-independent measure for each selected metric (Ginestet et al. 2011; Hosseini et al. 2012; Van Den Heuvel et al. 2017). Three graph metrics were estimated from connectivity matrices with sparsity levels in the range of 5% to 30% (with an increment of 1%), ensuring the conditions of connectedness (Fornito et al. 2010) and small-worldness (Watts and Strogatz 1998). The AUC of the threshold-related profile was calculated and extracted for each EEG frequency band and for each participant. The average CC and the CPL were calculated to evaluate the global level of segregation and integration of the EEG connectome (Rubinov and Sporns 2010). In the context of functional connectivity, higher average CC reflects the tendency of neighbouring nodes to form functional clusters, thereby supporting the establishment of highly synchronised units with elevated within-unit information exchange (Rubinov and Sporns 2010; Sporns 2016; Cohen and D’Esposito 2016; Zippo et al. 2018). Lower CPL values are consistent with greater functional integration, reflecting a network topology in which distant brain regions show stronger statistical interdependencies (Vuksanović and Hövel 2014; Has Silemek et al. 2024).

In addition, we extracted the SW, a summary metric obtained from the ratio of the normalised CC and CPL, useful to describe the balance between local segregation and global integration of the functional connectome (Watts and Strogatz 1998; Liao et al. 2017). A SW index larger than 1 was required to ensure the small-worldness of the EEG connectome. All topological metrics were calculated using the functions “*threshold_proportional”* (to threshold the connectivity matrices), averaging the output of *clustering_coef_wu* (to calculate average CC), *charpath* (to calculate CPL) and *randmio_und* (to generate a random network, preserving the degree distribution). All functions are embedded within the Brain Connectivity Toolbox (BCT, http://www.brainconnectivity-toolbox.net/).

### Statistical analysis

All data were analysed using MATLAB (The MathWorks, Inc., Natick, MA, United States) version R2023a. A statistically significant p-value was set at 0.05 for all statistical analyses. Partial Spearman correlations were computed to evaluate the pairwise relationship between the scores of the 11 neuropsychological tests and the values of the three graph-theoretical metrics (CC, CPL, SW), while controlling for age, years of education, and sex. To assess the robustness of the Spearman correlation results, we performed a bootstrap analysis using 1,000 resamples with replacement, calculating the correlation coefficient for each bootstrap sample and the corresponding 95% confidence intervals.

Scatterplots with trend lines were generated for the significant correlations to illustrate the distributions and the trends in the data. For the trend line, we applied both ordinary (OLS) and weighted (robust) regression methods to also check whether the presence of outliers would affect the results.

In addition, separately for each graph-theoretical metric, we performed a multivariate analysis to address frequency-related multicollinearity and to assess the extent to which each EEG band contributed to explaining the score on the neuropsychological tests, showing statistically significant correlations across multiple EEG bands. A principal component analysis (PCA) was applied, including the band-specific estimates of the graph metrics, and the minimum number of principal components was retained to ensure 95% explained variance. The resulting principal component scores were then used as independent variables in a generalised linear model (GLM) applied to the neuropsychological scores. This model, which included age, sex, and years of education as covariates, identified at least one significant correlation with the graph-theoretical metric. This analysis was designed to assess how combinations of frequency-dependent PCA components contributed to variability in neuropsychological performance.

To identify influential observations in GLM, the Cook’s distance analysis (Cook 1977) was performed. Observations with Cook’s distance values exceeding three times the mean Cook’s distance were flagged as potentially influential, and GLM estimates were compared with and without these observations. Corrections for multiple comparisons were based on the false discovery rate (FDR) method using the Benjamini–Hochberg procedure with a significance threshold of α = 0.05. FDR correction was applied separately within each EEG frequency band. For each band, the correction was performed across all 33 univariate correlations (11 neuropsychological scores × 3 graph-theoretical metrics). The same FDR correction procedure was applied to the statistical tests performed in the multivariate analyses.

## Results

The median values and the corresponding interquartile range (IQR) of the cognitive and social cognition scores are reported in Table 1. Overall, these scores indicated high performance levels with relatively restricted variability as indicated by the narrow IQRs across most measures.

The same descriptive information for the graph-theoretical metrics across the seven EEG frequency bands is reported in Table 2, showing a comparable median level and IQRs across the bands.

**Table 1:** Cognitive and social cognition scores (median, IQR)

Neuropsychological scores	Possible range	Observed range	Median (IQR)
MoCA	0–30	24–30	28 (28–30)
ESCoT_COGN_ToM	0–24	13–22	19 (17–19.5)
ESCoT_AFF_ToM	0–24	10–24	21 (19–23)
ESCoT_INTER	0–24	14–22	18 (17–19.5)
ESCoT_INTRA	0–24	19–24	23.5 (23–24)
ESCoT_TOTAL	0–96	63–87	81.5 (78.5–84)
QCAE_COGN	0–76	48–71	59 (53–64)
QCAE_AFF	0–48	26–47	36.5 (31–39.5)
SNQ_BREAK	0–12	7–11	10 (9–11)
SNQ_OVER	0–10	4–10	8 (7–9)
SNQ_TOTAL	0–22	14–21	18 (16–19)

The possible score range, the observed score range within the study sample, and the median value with the corresponding interquartile range (IQR; i.e., the distance between the 25th and 75th percentiles) are reported for the MoCA and the social cognition measures

*MoCA* Montreal Cognitive Assessment, *ESCoT* Edinburgh Social Cognition Test, *ToM* Theory of Mind, *COGN* Cognitive, *AFF* Affective, *INTER* Interpersonal norm, *INTRA* Intrapersonal norm, *QCAE* Questionnaire of Cognitive and Affective Empathy, *SNQ* Social Norm Questionnaire,* OVER* Overadhere score

**Table 2:** Resting-state EEG network metrics (CC, CPL, SW) by frequency band (median, IQR)

EEG Frequency bands	CC	CPL	SW
Delta	0.277 (0.252–0.294)	5.534 (5.110–6.030)	1.397 (1.280–1.492)
Theta	0.268 (0.248–0.292)	5.702 (5.315–6.117)	1.345 (1.266–1.495)
Alpha-1	0.275 (0.255–0.311)	5.616 (4.878–6.064)	1.279 (1.194–1.388)
Alpha-2	0.268 (0.257–0.307)	5.729 (4.872–6.110)	1.294 (1.212–1.451)
Beta-1	0.261 (0.249–0.299)	5.846 (5.092–6.274)	1.315 (1.264–1.504)
Beta-2	0.264 (0.245–0.284)	5.852 (5.442–6.211)	1.351 (1.251–1.567)
Gamma	0.266 (0.250–0.281)	5.768 (5.517–6.075)	1.393 (1.295–1.548)

The median value and IQR, i.e., the distance between the 25th and 75th percentiles of the distribution of values, are reported for the three graph-theoretical metrics, i.e., clustering coefficient (CC), characteristic path length (CPL) and small-world index (SW), in the seven canonical EEG frequency bands, i.e., delta: 2–4 Hz, theta: 4–8 Hz, alpha-1: 8–10.5 Hz, alpha-2: 10.5–13 Hz, beta-1: 13–20 Hz, beta-2: 20–30 Hz and gamma: 30–40 Hz.

### Correlation analysis

Intercorrelations among cognitive and social cognition measures are reported in Supplementary Table S1. ESCoT_INTRA was not significantly associated with MoCA, ESCoT_COGN_ToM, ESCoT_AFF_ToM, ESCoT_INTER, QCAE_COGN, QCAE_AFF, SNQ_BREAK, SNQ_OVER, or SNQ_TOTAL (all *p* > 0.05). The Spearman correlations between the 11 neuropsychological tests (MoCA and social cognition) and each graph metric were performed separately for the seven EEG frequency bands; results are reported in Tables 3, 4 and 5 for the CC, CPL and SW, respectively. Table values report ρ and uncorrected p-values; statistically significant correlations (FDR-corrected) are highlighted in bold. The bootstrapped 95% CIs are fully reported in Supplementary Tables S2-S4.

Regarding the CC, two negative correlations with the ESCoT_INTRA score were significant: one in the theta band (ρ = −0.642, 95% CI [−0.827, −0.300], *p* = 0.03, FDR-corrected) and one in the alpha-2 band (ρ = −0.616, 95% CI [−0.802, −0.305], *p* = 0.04, FDR-corrected).

Regarding the CPL, three positive correlations with the ESCoT_INTRA score were significant: in the theta band (ρ = 0.687, 95% CI [0.400, 0.852] *p* = 0.01, FDR-corrected), in the alpha-1 band (ρ = 0.642, 95% CI [0.372, 0.812], *p* = 0.03, FDR-corrected), and in the alpha-2 band (ρ = 0.710, 95% CI [0.456, 0.826], *p* = 0.01, FDR-corrected).

No significant associations were observed for SW (*p* > 0.05 for all).

**Fig. 1:**
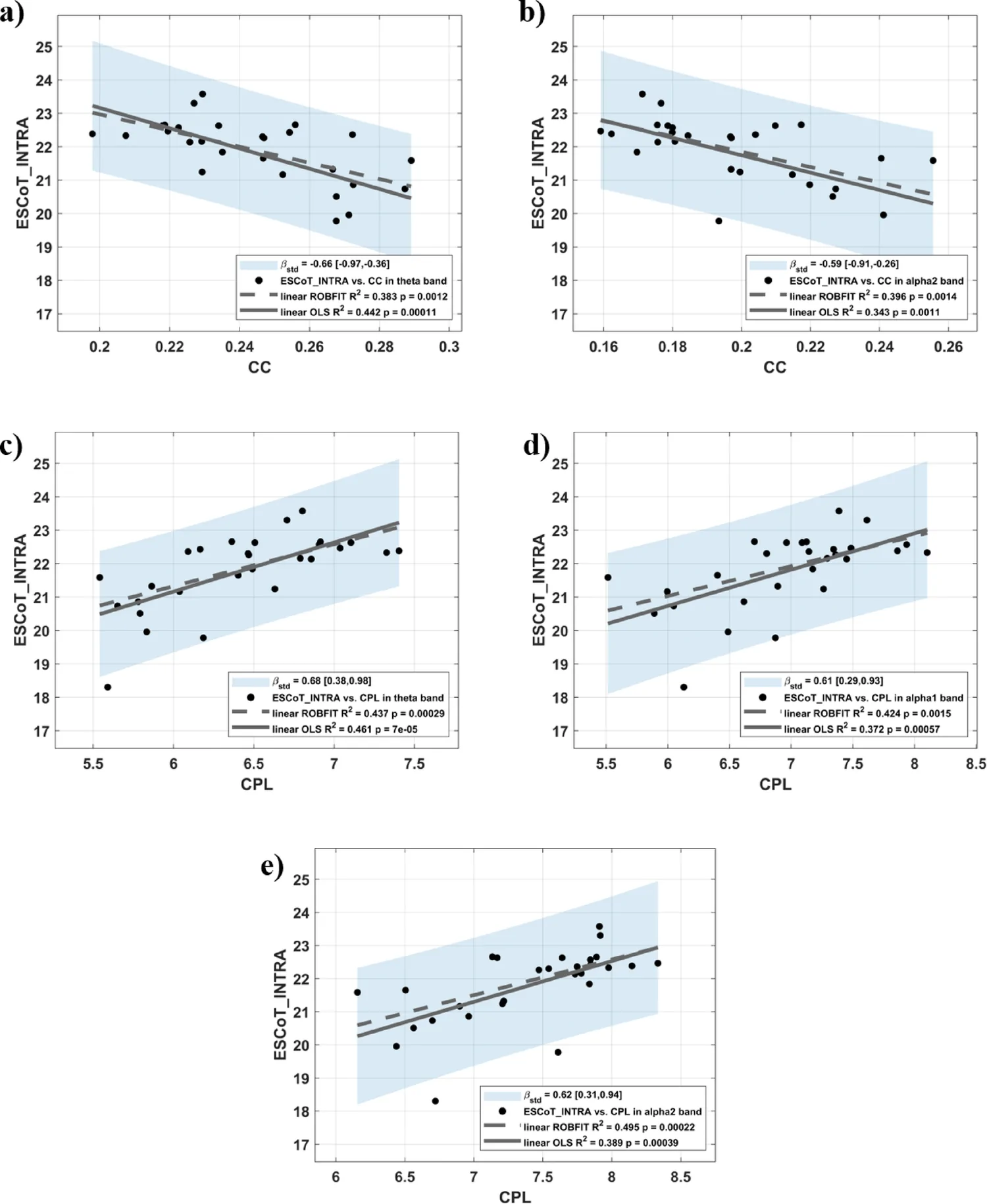
Scatterplots of associations between the intrapersonal norm score (ESCoT_INTRA) and network metrics that survived FDR correction. Only associations surviving FDR correction are displayed. Individual observations, fitted lines obtained using ordinary least squares (OLS) and robust regression, and 95% confidence intervals are shown. **a** The negative correlation between ESCoT_INTRA and the CC in the theta band (R-squared robust fit = 0.383, R-squared OLS = 0.442, p-value = 0.001**); **b** The negative correlation between ESCoT_INTRA and the CC in the alpha-2 band (R-squared robust fit = 0.396, R-squared OLS = 0.343, p-value = 0.0014**); **c** The positive correlation between ESCoT_INTRA and the CPL in the theta band (R-squared robust fit = 0.437, R-squared OLS = 0.461, p-value = 0.003**); **d** The positive correlation between ESCoT_INTRA and the CPL in the alpha-1 band (R-squared robust fit = 0.424, R-squared OLS = 0.372, p-value = 0.0015**); **e** The positive correlation between ESCoT_INTRA and the CPL in the alpha-2 band (R-squared robust fit = 0.495, R-squared OLS = 0.389, p-value = 0.0002**) [*p < 0.05; **p < 0.01]

Figure 1 illustrates the significant associations surviving FDR correction as scatterplots, including individual observations, fitted lines obtained using ordinary least squares (OLS) and robust regression, 95% confidence intervals around the fitted regression lines, coefficients of determination, and p-values. Robust and OLS regression analyses yielded nearly similar results, with no meaningful differences in the direction, magnitude, or statistical significance of the estimated associations, suggesting that the associations were not driven by influential observations. Comparisons between OLS and robust regression estimates for the statistically significant associations are reported in Supplementary Tables S5-S9.

**Table 3:** Spearman correlations between CC and neuropsychological scores across EEG bands (ρ, p)

Clustering Coefficient (CC)							
Cognitive score	Delta ρ-val (p-val)	Theta ρ-val (p-val)	Alpha-1 ρ-val (p-val)	Alpha-2 ρ-val (p-val)	Beta-1 ρ-val (p-val)	Beta-2 ρ-val (p-val)	Gamma ρ-val (p-val)
MoCA	−0.159 (0.445)	−0.015 (0.943)	0.145 (0.487)	−0.002 (0.993)	0.005 (0.980)	−0.179 (0.390)	−0.214 (0.304)
ESCoT_COGN_ToM	0.461 (0.020*)	0.326 (0.111)	0.222 (0.284)	0.136 (0.515)	0.243 (0.241)	0.330 (0.106)	0.178 (0.395)
ESCoT_AFF_ToM	0.173 (0.406)	0.359 (0.077)	0.436 (0.029*)	0.304 (0.139)	0.263 (0.203)	0.074 (0.724)	−0.163 (0.436)
ESCoT_INTER	0.344 (0.091)	0.105 (0.616)	−0.032 (0.877)	0.063 (0.764)	0.143 (0.496)	0.192 (0.356)	0.176 (0.398)
ESCoT_INTRA	−0.571 (0.0028**)	**−0.642** (0.0005**)	−0.562 (0.0034**)	**−0.616** (0.001**)	−0.559 (0.004**)	−0.292 (0.156)	−0.109 (0.601)
ESCoT_TOTAL	0.242 (0.243)	0.168 (0.422)	0.179 (0.389)	0.125 (0.552)	0.146 (0.484)	0.136 (0.516)	−0.034 (0.872)
QCAE_COGN	0.063 (0.763)	0.052 (0.804)	0.111 (0.597)	0.028 (0.891)	−0.021 (0.921)	−0.077 (0.712)	−0.035 (0.867)
QCAE_AFF	−0.099 (0.636)	−0.131 (0.532)	−0.087 (0.679)	0.048 (0.820)	−0.036 (0.863)	0.136 (0.393)	−0.259 (0.211)
SNQ_BREAK	−0.132 (0.530)	−0.191 (0.361)	0.007 (0.972)	0.056 (0.791)	−0.067 (0.747)	−0.228 (0.271)	−0.109 (0.603)
SNQ_OVER	0.042 (0.840)	0.225 (0.279)	0.298 (0.147)	0.129 (0.537)	0.062 (0.765)	0.088 (0.675)	0.015 (0.941)
SNQ_TOTAL	−0.182 (0.382)	−0.055 (0.793)	0.173 (0.408)	0.084 (0.688)	−0.056 (0.789)	−0.115 (0.584)	−0.095 (0.651)

Spearman’s correlations between the mean CC, in the seven EEG frequency bands of interest (delta: 2–4 Hz; theta: 4–8 Hz; alpha-1: 8–10.5 Hz; alpha-2: 10.5–13 Hz; beta-1: 13–20 Hz; beta-2: 20–30 Hz; gamma: 30–40 Hz) and the neuropsychological scores (MoCA and social cognition measures). Correlation coefficients and corresponding uncorrected p-values are reported. Significant correlations after FDR correction are reported in bold. [*p < 0.05; **p < 0.01]

*MoCA* Montreal Cognitive Assessment,* ESCoT* Edinburgh Social Cognition Test, *ToM* Theory of Mind, *COGN* Cognitive, *AFF* Affective, *INTER* Interpersonal norm, *INTRA* Intrapersonal norm, *QCAE* Questionnaire of Cognitive and Affective Empathy, *SNQ* Social Norm Questionnaire, *OVER* Overadhere score

**Table 4:** Spearman correlations between CPL and neuropsychological scores across EEG bands (ρ, p)

Characteristic Path Length (CPL)							
Cognitive score	Delta ρ-val (p-val)	Theta ρ-val (p-val)	Alpha-1 ρ-val (p-val)	Alpha-2 ρ-val (p-val)	Beta-1 ρ-val (p-val)	Beta-2 ρ-val (p-val)	Gamma ρ-val (p-val)
MoCA	0.151 (0.470)	−0.007 (0.974)	−0.094 (0.654)	−0.018 (0.931)	0.010 (0.959)	0.115 (0.582)	0.119 (0.570)
ESCoT_COGN_ToM	−0.407 (0.043*)	−0.359 (0.077)	−0.204 (0.326)	−0.155 (0.459)	−0.314 (0.125)	−0.390 (0.053)	−0.264 (0.200)
ESCoT_AFF_ToM	−0.185 (0.375)	−0.315 (0.124)	−0.388 (0.054)	−0.261 (0.207)	−0.266 (0.197)	−0.117 (0.574)	0.105 (0.614)
ESCoT_INTER	−0.416 (0.038*)	−0.158 (0.449)	0.020 (0.925)	−0.118 (0.572)	−0.196 (0.346)	−0.226 (0.276)	−0.267 (0.196)
ESCoT_INTRA	0.587 (0.002**)	**0.687** (0.0001**)	**0.642** (0.0005**)	**0.710** (0.00007**)	0.602 (0.001**)	0.329 (0.107)	0.221 (0.286)
ESCoT_TOTAL	−0.262 (0.205)	−0.168 (0.420)	−0.131 (0.533)	−0.107 (0.609)	−0.203 (0.328)	−0.212 (0.308)	−0.064 (0.759)
QCAE_COGN	−0.003 (0.988)	−0.048 (0.818)	−0.070 (0.739)	−0.047 (0.822)	0.001 (0.995)	0.044 (0.834)	−0.048 (0.818)
QCAE_AFF	0.228 (0.272)	0.113 (0.590)	0.028 (0.891)	−0.072 (0.733)	−0.036 (0.863)	0.116 (0.579)	0.189 (0.364)
SNQ_BREAK	0.129 (0.538)	0.150 (0.474)	0.042 (0.841)	−0.143 (0.495)	0.057 (0.785)	0.149 (0.476)	0.014 (0.946)
SNQ_OVER	−0.108 (0.606)	−0.256 (0.216)	−0.403 (0.045*)	−0.192 (0.358)	−0.098 (0.641)	−0.097 (0.642)	−0.001 (0.997)
SNQ_TOTAL	0.139 (0.504)	−0.006 (0.975)	−0.248 (0.230)	−0.224 (0.279)	−0.016 (0.937)	−0.001 (0.996)	0.010 (0.959)

Spearman’s correlations between the CPL, in the seven EEG frequency bands of interest (delta: 2–4 Hz; theta: 4–8 Hz; alpha-1: 8–10.5 Hz; alpha-2: 10.5–13 Hz; beta-1: 13–20 Hz; beta-2: 20–30 Hz; gamma: 30–40 Hz) and the neuropsychological scores (MoCA and social cognition measures). Correlation coefficients and corresponding uncorrected p-values are reported. Significant correlations after FDR correction are reported in bold. [*p < 0.05; **p < 0.01]

*MoCA* Montreal Cognitive Assessment,* ESCoT* Edinburgh Social Cognition Test, *ToM* Theory of Mind, *COGN* Cognitive, *AFF* Affective, *INTER* Interpersonal norm, *INTRA* Intrapersonal norm, *QCAE* Questionnaire of Cognitive and Affective Empathy, *SNQ* Social Norm Questionnaire, *OVER* Overadhere score

**Table 5:** Spearman correlations between SW and neuropsychological scores across EEG bands (ρ, p)

Small-World index (SW)							
Cognitive score	Delta ρ-val (p-val)	Theta ρ-val (p-val)	Alpha-1 ρ-val (p-val)	Alpha-2 ρ-val (p-val)	Beta-1 ρ-val (p-val)	Beta-2 ρ-val (p-val)	Gamma ρ-val (p-val)
MoCA	0.008 (0.971)	−0.031 (0.882)	0.037 (0.861)	0.114 (0.585)	0.095 (0.651)	0.070 (0.738)	0.270 (0.192)
ESCoT_COGN_ToM	−0.211 (0.310)	−0.218 (0.294)	−0.210 (0.312)	−0.069 (0.740)	−0.155 (0.459)	−0.153 (0.463)	−0.286 (0.165)
ESCoT_AFF_ToM	−0.305 (0.137)	−0.332 (0.104)	−0.151 (0.470)	−0.196 (0.346)	−0.218 (0.293)	−0.124 (0.553)	−0.091 (0.664)
ESCoT_INTER	0.127 (0.543)	0.146 (0.486)	0.084 (0.689)	−0.039 (0.853)	0.204 (0.327)	0.116 (0.580)	−0.041 (0.845)
ESCoT_INTRA	0.085 (0.685)	0.087 (0.677)	−0.035 (0.868)	−0.046 (0.827)	0.202 (0.332)	0.216 (0.298)	−0.002 (0.991)
ESCoT_TOTAL	−0.175 (0.401)	−0.183 (0.381)	−0.101 (0.632)	−0.126 (0.548)	−0.014 (0.946)	0.019 (0.927)	−0.174 (0.404)
QCAE_COGN	−0.046 (0.824)	0.001 (0.997)	−0.041 (0.844)	−0.028 (0.894)	0.204 (0.326)	0.204 (0.328)	0.115 (0.582)
QCAE_AFF	−0.061 (0.773)	−0.071 (0.735)	−0.111 (0.595)	0.059 (0.780)	0.048 (0.818)	0.144 (0.493)	0.322 (0.116)
SNQ_BREAK	0.045 (0.830)	−0.035 (0.868)	−0.012 (0.956)	−0.037 (0.858)	0.023 (0.911)	−0.028 (0.894)	−0.088 (0.672)
SNQ_OVER	−0.211 (0.311)	−0.230 (0.267)	−0.142 (0.497)	−0.067 (0.750)	−0.044 (0.833)	0.0002 (0.999)	−0.342 (0.094)
SNQ_TOTAL	−0.291 (0.157)	−0.327 (0.110)	−0.219 (0.293)	−0.162 (0.438)	−0.074 (0.722)	−0.042 (0.840)	−0.336 (0.100)

Spearman’s correlations between the small-world index (SW), in the seven EEG frequency bands of interest (delta: 2–4 Hz; theta: 4–8 Hz; alpha-1: 8–10.5 Hz; alpha-2: 10.5–13 Hz; beta-1: 13–20 Hz; beta-2: 20–30 Hz; gamma: 30–40 Hz) and the neuropsychological scores (MoCA and social cognition measures). Correlation coefficients and corresponding uncorrected p-values are reported. Significant correlations after FDR correction are reported in bold. [*p < 0.05; **p < 0.01]

*MoCA* Montreal Cognitive Assessment,* ESCoT* Edinburgh Social Cognition Test, *ToM* Theory of Mind, *COGN* Cognitive, *AFF* Affective, *INTER* Interpersonal norm, *INTRA* Intrapersonal norm, *QCAE* Questionnaire of Cognitive and Affective Empathy, *SNQ* Social Norm Questionnaire, *OVER* Overadhere score

### Principal component analysis (PCA)

A PCA was performed separately for each graph theoretical metric to investigate the joint contribution across five EEG frequency bands that showed significant associations in the univariate analysis. For each graph metric, PCA revealed the following percentages of explained variance:

- CC (delta, theta, alpha-1, alpha-2, beta-1): 82.46%, 10.42%, 4.82%, 1.86%, 0.42%.
- CPL (delta, theta, alpha-1, alpha-2, beta-1): 83.32%, 9.70%, 4.77%, 1.68%, 0.51%.
- SW (delta, theta, alpha-1, alpha-2, beta-1): 82.00%, 10.46%, 5.35%, 1.73%, 0.44%.

We retained the first three components (pc1, pc2, pc3) because together they accounted for 95% of the cumulative explained variance. The loadings of the original frequency-derived variables on all five components were also calculated and reported in the Supplementary Tables S10-S12, separately for CC, CPL and SW.

The first three components showed similar loading patterns across the three graph-theoretical metrics. Frequency-band variables contributed relatively evenly to pc1 across all three graph-theoretical metrics, with contributions observed across multiple low-to-mid frequency bands. At the same time, *pc2* was positively and prominently influenced by the delta band component for all three metrics. The pc3 showed a higher-frequency loading pattern. For CC and CPL, it was mainly characterised by negative alpha-1 and positive beta-1 loadings, whereas for SW it was mainly characterised by positive alpha-2 and negative beta-1 loadings. In Supplementary Figures S2-S4, we displayed 2D PCA biplots to illustrate the frequency-related loadings of the first three principal components separately for each graph-theoretical metric.

**Fig. 2:**
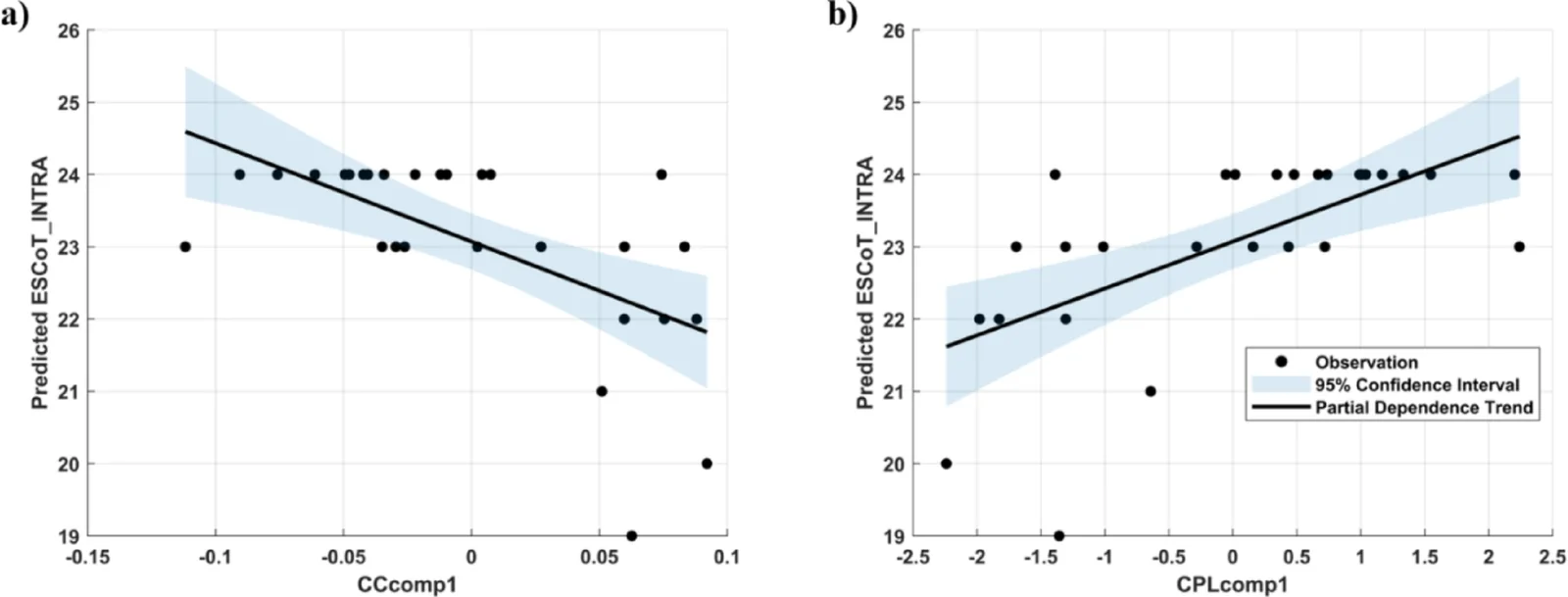
Partial dependence plots showing the relationship between the intrapersonal norm score (ESCoT_INTRA) and the first principal component (*pc1*), while controlling for the remaining principal components (*pc2*, *pc3*), age, sex, and years of education. Panels display the associations between model-predicted ESCoT_INTRA scores and pc1 derived from **a** CC (β = −13.61, t-stat = −3.8758, *p* = 0.0008, FDR-corrected *p* = 0.020) and **b** CPL (β = 0.648, t-stat = 4.1021, *p* = 0.0005, FDR-corrected *p* = 0.020). Individual observations, fitted regression lines, and 95% confidence intervals are shown

Finally, the GLM-based regression of ESCoT_COGN_ToM, ESCoT_AFF_ToM, ESCoT_INTER, ESCoT_INTRA and SNQ_OVER on the selected principal components (as predictors), controlling for age, sex and years of education variables, showed significant associations between ESCoT_INTRA and the first principal component derived from CC (t-stat = −3.8758, *p* = 0.0008, FDR-corrected *p* = 0.020) and from CPL (t-stat = 4.1021, *p* = 0.0005, FDR-corrected *p* = 0.020). Figure 2 displays partial dependence plots visualising these significant associations. Additional information on the significant GLM analyses and influence diagnostics is reported in Supplementary Figure S1 and Supplementary Tables S13–S14. Cook’s distance analyses identified potentially influential observations; however, GLM estimates were highly similar before and after their exclusion. Therefore, the full-sample GLM models were retained as the final analyses.

## Discussion

The present study aimed at investigating the association between social cognition and brain connectivity, and examining whether inter-individual differences relate to the topological organisation of the resting-state EEG functional connectome across canonical frequency bands.

Our findings showed a selective association between intrapersonal norm processing and the topological properties of EEG networks, consistent with prior evidence linking global network topology and inter-individual variability in cognitive performance (Van Den Heuvel et al. 2009; Langer et al. 2012; Finn et al. 2015). We found that higher intrapersonal norm scores covaried with lower CC and longer CPL, most consistently in the theta and alpha bands. These associations remained significant after controlling for demographic variables and were supported by complementary multivariate analyses summarising frequency-dependent graph-theoretical properties. These multivariate findings suggest that individual differences in intrapersonal norm processing may not depend on the topology of a single EEG oscillation, but rather on the combined contribution of multiple low-to-mid frequency bands (Mwilambwe-Tshilobo et al. 2023).

Interpreting graph metrics in the context of cognitive network neuroscience provides a useful framework for understanding these findings and assessing how topological brain organisation relates to cognitive performance. Global CC indexes the average tendency of neighbouring regions to promote local functional synchronisation, whereas CPL indexes global (dis)integration (Watts and Strogatz 1998; De Haan et al. 2009; Rubinov and Sporns 2010; Sporns 2016). While resting-state graph metrics cannot be interpreted as direct markers of specific computational processes, the observed association between lower CC and longer CPL with higher intrapersonal norm scores is consistent with the hypothesis that coordination-intensive socio-cognitive operations may rely on a more spatially distributed organisational topology, characterised by reduced local clustering and a more distributed pattern of network organisation. These findings do not imply that more integrated network configurations (i.e., shorter path lengths) more uniformly support cognitive performance (Langer et al. 2012; Cohen and D’Esposito 2016). While such configurations appear advantageous in domains such as intelligence and working memory, accumulating evidence indicates that the relationship between network efficiency and behaviour is not strictly monotonic. For example, resting-state EEG evidence has shown that longer CPL in the alpha band is positively associated with non-verbal intelligence (Zakharov et al. 2020), whereas a resting-state fMRI study has reported that higher global efficiency is associated with slower planning performance (Vriend et al. 2020). Taken together, these findings support the view that the relationship between connectome topology and cognitive performance is domain-dependent rather than universally monotonic. While certain cognitive domains may benefit from highly integrated configurations, socio-cognitive functions such as intrapersonal norm reasoning may rely on a distinct balance between segregation and integration. This perspective further refines existing models of network efficiency by emphasising the functional specificity of topological–behavioural associations, contributing to a more nuanced account of connectome efficiency, in which optimal topological configurations are context-sensitive rather than universally advantageous.

The present findings extend network efficiency models to the socio-cognitive domain and highlight the importance of domain-sensitive interpretations of connectome topology. Intrapersonal norm reasoning in the ESCoT requires the engagement of several heterogeneous processes, including the perception and interpretation of the social scene, the simulation of one’s own behaviour in that context, the activation of internalised moral and social-knowledge representations, and the evaluation of behavioural appropriateness. These processes may operate in parallel rather than sequentially, and therefore may not necessarily align with network configurations characterised by extreme segregation or high global integration. Configurations characterised by pronounced segregation or maximal global integration may not optimally support the simultaneous coordination of perceptual, self-referential, and normative information streams. Instead, a more diffuse and flexible topological organisation (i.e., concomitant reduced local synchronisation and longer inter-regional communication) may facilitate the flexible interplay among heterogeneous processes required for intrapersonal normative reasoning. Although the present cross-sectional design does not permit mechanistic conclusions, the observed topological pattern is consistent with accounts emphasising the importance of balanced segregation and integration for complex, coordination-intensive cognition.

A further consideration concerns the specificity of the associations observed for the intrapersonal norm component of the ESCoT. Rather than reflecting a process that is uniquely self-related, intrapersonal norm reasoning requires individuals to evaluate how they themselves would behave within a given social context while considering social rules and behavioural appropriateness. Similar self-referential and simulation-based processes have also been implicated in theory of mind (Spreng and Grady 2010). Intrapersonal norm reasoning likely involves the interaction of multiple processes, including perspective-taking, self-referential evaluation, behavioural regulation, and normative knowledge. Such a multidimensional profile of information processing may be especially sensitive to global patterns of resting-state connectome organisation (Van Overwalle 2009; Reniers et al. 2011; Bzdok et al. 2012). This pattern of information processing and communication plausibly depends on the interaction among multiple distributed neural systems, which is consistent with our finding that a less segregated and less tightly integrated topology of the functional connectome is associated with higher intrapersonal norm performance. Interestingly, intrapersonal norm processing was not significantly associated with the other measures of social cognition included in the present study (Supplementary Table S1), suggesting that it may reflect a partially distinct aspect, not fully shared with theory of mind, empathy, or explicit social norm knowledge.

The observed frequency-dependent pattern is consistent with previous studies linking slower oscillations to internally oriented cognition, cognitive control, and the coordination of large-scale interactions (Buzsáki 2006; Fries 2015; Jiang et al. 2024). Theta oscillations, particularly in frontal regions, have been linked to executive monitoring, emotional regulation, and rule maintenance (Hsieh and Ranganath 2014; Cavanagh and Frank 2014; Adamczyk and Wyczesany 2023), whereas alpha rhythms support the gating of internally oriented processing and the inhibition of task-irrelevant information (Benedek et al. 2011; Klimesch 2012; Fries 2015; Riddle et al. 2020).

In addition to this general pattern, the current findings revealed partially overlapping, yet distinguishable, associations between network topology and the use and understanding of intrapersonal norms across frequency bands. Both average CC and CPL were related to intrapersonal norm scores in theta and alpha-2 bands, whereas an additional effect in alpha-1 emerged only for CPL. However, these findings should not be interpreted as evidence of strict frequency specificity. Rather, they suggest that theta and alpha band oscillations may jointly contribute to large-scale network organisation relevant for normative self-reflection.

Taken together, this pattern suggests that theta and alpha band topological properties may index complementary aspects of large-scale neural coordination relevant for intrapersonal norm processing, rather than reflecting isolated frequency-specific mechanisms.

Several limitations of the present study should be acknowledged. First, the relatively modest sample size limits statistical power and may inflate effect-size estimates; replication in larger cohorts will be important to establish the robustness of the observed associations. In addition, several behavioural measures showed restricted score distributions and potential ceiling effects, which may have reduced sensitivity to detect weaker associations. Second, the thresholding strategy introduces methodological variability (Van Wijk et al. 2010). Even though the cost-integration method was applied to overcome the limitations of a priori selected threshold, future studies should compare alternative thresholding procedures to assess the stability of the reported effects (Dimitriadis et al. 2017). Moreover, although source reconstruction partially reduces the influence of volume conduction and spatial leakage, these effects cannot be eliminated and may still influence functional connectivity estimates and derived graph-theoretical metrics (Bastos and Schoffelen 2016). Future studies should therefore evaluate the robustness of the present findings across alternative connectivity estimators designed to reduce the influence of zero-lag coupling, such as the phase lag index, weighted phase lag index, and imaginary coherence. Third, we explored associations with global topological properties, consistent with the view that social cognition emerges from the coordinated activity of large-scale neural systems. However, the exploratory nature of the present study and the limited sample size did not allow us to identify the specific regions or subnetworks primarily driving these associations. Future studies should therefore complement global graph-theoretical metrics with network-based approaches (e.g., analyses on canonical large-scale functional systems; Yeo et al. 2011) to clarify which, and to what extent, functional brain networks drive these associations (Chan et al. 2014). Finally, the cross-sectional design of the present study precludes causal inference. Furthermore, because the present study relied on resting-state EEG rather than task-based recordings, the observed associations should not be interpreted as evidence that the identified topological configuration directly supports intrapersonal norm processing. Rather, they suggest that individual differences in intrapersonal norm processing may be associated with intrinsic network organisation. In addition, the moderate test–retest reliability of resting-state EEG measures (Näpflin et al. 2007; Fraschini et al. 2016; Kuntzelman and Miskovic 2017) may limit their suitability as stable proxies for social cognitive trait-level differences. Future studies combining longitudinal designs, multimodal approaches, and task-based paradigms will therefore be necessary to determine whether the present findings reflect stable individual characteristics, task-related neural dynamics, or an interaction between the two, and whether the identified resting-state topological properties track changes in social cognition over time or under experimental manipulation. Importantly, several lines of evidence indicate that social cognition abilities can be modified through behavioural interventions, social cognitive training or neurostimulation (e.g., Kurtz and Richardson 2012; Santiesteban et al. 2012; Young et al. 2010), supporting the notion that the topology of the resting-state functional (EEG-derived) connectome may relate to dynamic changes in social cognition performance.

## Conclusion

The present findings provide novel, yet preliminary, evidence that intrapersonal processing of social norms in healthy young adults is associated with specific features of resting-state EEG topology. Lower clustering and longer CPL within theta and alpha frequency bands are linked to better performance in normative self-reflection, suggesting that complex socio-cognitive processes may benefit from a more distributed and flexible network organisation relying on slower oscillatory rhythms. By bridging multiband EEG connectomics with behavioural measures, this study promotes the value of EEG graph-theoretical approaches for capturing individual variability in cognitive functions, contributing to the emerging field of social cognitive network neuroscience and offering testable hypotheses for future work.

## Supplementary Information

Below is the link to the electronic supplementary material.

Supplementary Material 1

## Data Availability

The data that support the findings of this study are available from the corresponding author upon reasonable request.
