# Supplementary material for: Resting-state EEG connectome topology and social norm processing in healthy young adults
Source: Brain Struct Funct. 2026 Jul 30;231(7):110. doi: 10.1007/s00429-026-03167-9 (PMC13424021; doi:10.1007/s00429-026-03167-9)
Supplement: Supplementary file 1 — Supplementary Material 1 [file 429_2026_3167_MOESM1_ESM.docx]

**Supplementary Materials**

**Table S1.** Intercorrelations among cognitive and social cognition measures.


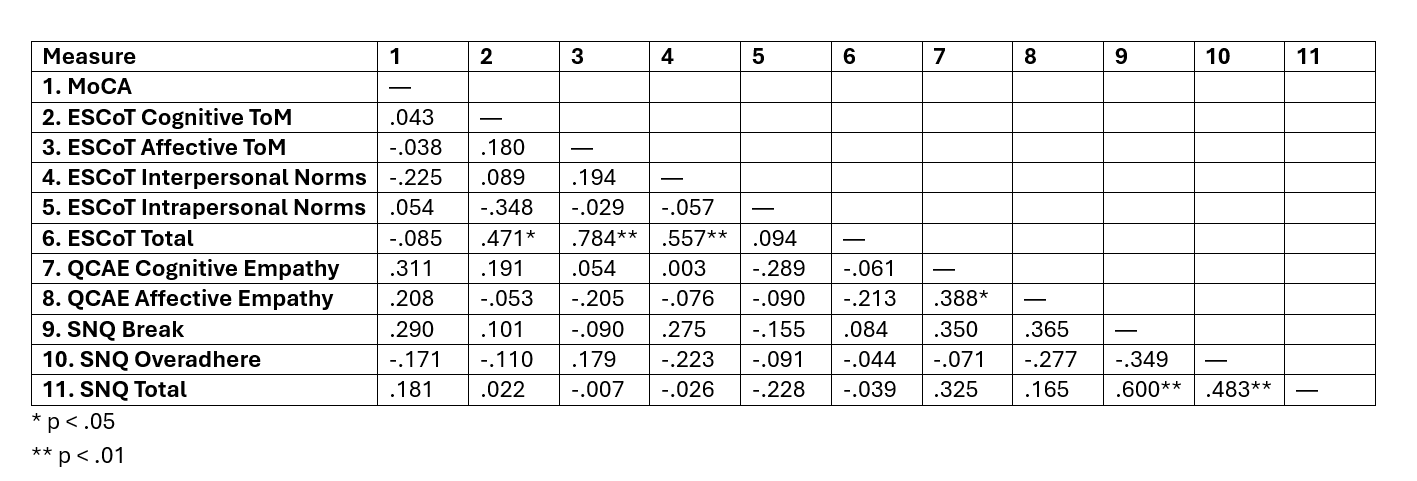


**Table S2.** Bootstrapped 95% confidence intervals for Spearman’s rank correlation coefficients between cognitive scores and clustering coefficient across EEG frequency bands.

| Clustering Coefficient (CC) | | | | | | | |
| --- | --- | --- | --- | --- | --- | --- | --- |
| Cognitive score | **Delta**  **ρ-val**  **[95% CI]** | **Theta**  **ρ-val**  **[95% CI]** | **Alpha-1**  **ρ-val**  **[95% CI]** | **Alpha-2**  **ρ-val**  **[95% CI]** | **Beta-1**  **ρ-val**  **[95% CI]** | **Beta-2**  **ρ-val**  **[95% CI]** | **Gamma**  **ρ-val**  **[95% CI]** |
| MoCA | -0.159  [-0.47,0.28] | -0.015  [-0.39,0.37] | 0.145  [-0.36,0.51] | -0.002  [-0.43,0.42] | 0.005  [-0.37,0.40] | -0.179  [-0.62,0.23] | -0.214  [-0.66,0.25] |
| ESCoT_COGN_ToM | 0.461*  [0.04,0.71] | 0.326  [-0.08,0.65] | 0.222  [-0.16,0.58] | 0.136  [-0.18,0.42] | 0.243  [-0.13,0.54] | 0.330  [-0.09,0.66] | 0.178  [-0.24,0.56] |
| ESCoT_AFF_ToM | 0.173  [-0.27,0.59] | 0.359  [-0.09,0.71] | 0.436 *  [0.06,0.69] | 0.304  [-0.15,0.65] | 0.263  [-0.26,0.69] | 0.074  [-0.44,0.51] | -0.163  [-0.68,0.36] |
| ESCoT_INTER | 0.344  [-0.07,0.72] | 0.105  [-0.31,0.57] | -0.032  [-0.43,0.39] | 0.063  [-0.33,0.53] | 0.143  [-0.30,0.63] | 0.192  [-0.24,0.73] | 0.176  [-0.25,0.65] |
| ESCoT_INTRA | -0.571 **  [-0.75,-0.19] | **-0.642 ****  **[-0.83,-0.30]** | -0.562 **  [-0.77,-0.22] | **-0.616 ****  **[-0.80,-0.30]** | -0.559 **  [-0.77,-0.24] | -0.292  [-0.62,0.20] | -0.109  [-0.50,0.35] |
| ESCoT_TOTAL | 0.242  [-0.17,0.64] | 0.168  [-0.25,0.58] | 0.179  [-0.18,0.52] | 0.125  [-0.29,0.51] | 0.146  [-0.33,0.59] | 0.136  [-0.37,0.58] | -0.034  [-0.56,0.45] |
| QCAE_COGN | 0.063  [-0.34,0.47] | 0.052  [-0.37,0.48] | 0.111  [-0.37,0.55] | 0.028  [-0.29,0.51] | -0.021  [-0.38,0.33] | -0.077  [-0.47,0.37] | -0.035  [-0.46,0.36] |
| QCAE_AFF | -0.099  [-0.52,0.30] | -0.131  [-0.53,0.36] | -0.087  [-0.51,0.48] | 0.048  [-0.43,0.40] | -0.036  [-0.43,0.36] | 0.136  [-0.54,0.21] | -0.259  [-0.59,0.19] |
| SNQ_BREAK | -0.132  [-0.51,0.31] | -0.191  [-0.53,0.25] | 0.007  [-0.38,0.39] | 0.056  [-0.37,0.47] | -0.067  [-0.44,0.38] | -0.228  [-0.58,0.19] | -0.109  [-0.46,0.25] |
| SNQ_OVER | 0.042  [-0.36,0.50] | 0.225  [-0.18,0.67] | 0.298  [-0.16,0.67] | 0.129  [-0.37,0.50] | 0.062  [-0.37,0.58] | 0.088  [-0.34,0.60] | 0.015  [-0.43,0.46] |
| SNQ_TOTAL | -0.182  [-0.51,0.25] | -0.055  [-0.36,0.44] | 0.173  [-0.22,0.57] | 0.084  [-0.29,0.59] | -0.056  [-0.40,0.40] | -0.115  [-0.48,0.34] | -0.095  [-0.51,0.38] |

Bootstrapped 95% confidence intervals for Spearman’s correlations between the mean clustering coefficient (CC), in the seven EEG frequency bands of interest (delta: 2–4 Hz; theta: 4–8 Hz; alpha-1: 8–10.5 Hz; alpha-2: 10.5–13 Hz; beta-1: 13–20 Hz; beta-2: 20–30 Hz; gamma: 30–40 Hz) and the neuropsychological scores (MoCA and social cognition measures). Correlation coefficients and bootstrapped 95% confidence intervals are reported. Asterisks indicate uncorrected statistical significance (*p < 0.05; **p < 0.01). Significant correlations after FDR correction are reported in bold.

MoCA= Montreal Cognitive Assessment; ESCoT= Edinburgh Social Cognition Test; ToM= Theory of Mind; COGN= Cognitive; AFF= Affective; INTER= Interpersonal norm; INTRA= Intrapersonal norm; QCAE= Questionnaire of Cognitive and Affective Empathy; SNQ= Social Norm Questionnaire; OVER= Overadhere score

**Table S3.** Bootstrapped 95% confidence intervals for Spearman’s rank correlation coefficients between cognitive scores and characteristic path length across EEG frequency bands.

| Characteristic Path Length (CPL) | | | | | | | |
| --- | --- | --- | --- | --- | --- | --- | --- |
| Cognitive score | **Delta**  **ρ-val**  **[95% CI]** | **Theta**  **ρ-val**  **[95% CI]** | **Alpha-1**  **ρ-val**  **[95% CI]** | **Alpha-2**  **ρ-val**  **[95% CI]** | **Beta-1**  **ρ-val**  **[95% CI]** | **Beta-2**  **ρ-val**  **[95% CI]** | **Gamma**  **ρ-val**  **[95% CI]** |
| MoCA | 0.151  [-0.30,0.51] | -0.007  [-0.41,0.37] | -0.094  [-0.46,0.38] | -0.018  [-0.44,0.40] | 0.010  [-0.39,0.39] | 0.115  [-0.27,0.59] | 0.119  [-0.29,0.57] |
| ESCoT_COGN_ToM | -0.407 *  [-0.69,-0.01] | -0.359  [-0.68,0.07] | -0.204  [-0.55,0.20] | -0.155  [-0.48,0.21] | -0.314  [-0.62,0.09] | -0.390  [-0.69,0.03] | -0.264  [-0.61,0.12] |
| ESCoT_AFF_ToM | -0.185  [-0.57,0.25] | -0.315  [-0.66,0.12] | -0.388  [-0.66,0.01] | -0.261  [-0.64,0.16] | -0.266  [-0.65,0.25] | -0.117  [-0.54,0.38] | 0.105  [-0.42,0.64] |
| ESCoT_INTER | -0.416 *  [-0.78,0.00] | -0.158  [-0.65,0.27] | 0.020  [-0.41,0.39] | -0.118  [-0.59,0.29] | -0.196  [-0.67,0.23] | -0.226  [-0.73,0.21] | -0.267  [-0.67,0.19] |
| ESCoT_INTRA | 0.587 **  [0.26,0.75] | **0.687** **  **[0.40,0.85]** | **0.642** **  **[0.37,0.81]** | **0.710** **  **[0.46,0.83]** | 0.602 **  [0.29,0.81] | 0.329  [-0.14,0.66] | 0.221  [-0.24,0.56] |
| ESCoT_TOTAL | -0.262  [-0.64,0.12] | -0.168  [-0.59,0.27] | -0.131  [-0.49,0.24] | -0.107  [-0.51,0.28] | -0.203  [-0.63,0.31] | -0.212  [-0.62,0.32] | -0.064  [-0.51,0.48] |
| QCAE_COGN | -0.003  [-0.42,0.42] | -0.048  [-0.47,0.38] | -0.070  [-0.56,0.39] | -0.047  [-0.45,0.37] | 0.001  [-0.39,0.37] | 0.044  [-0.41,0.45] | -0.048  [-0.42,0.39] |
| QCAE_AFF | 0.228  [-0.15,0.58] | 0.113  [-0.38,0.52] | 0.028  [-0.51,0.47] | -0.072  [-0.49,0.36] | -0.036  [-0.46,0.38] | 0.116  [-0.31,0.47] | 0.189  [-0.27,0.58] |
| SNQ_BREAK | 0.129  [-0.32,0.51] | 0.150  [-0.30,0.50] | 0.042  [-0.31,0.41] | -0.143  [-0.48,0.29] | 0.057  [-0.34,0.43] | 0.149  [-0.23,0.52] | 0.014  [-0.38,0.42] |
| SNQ_OVER | -0.108  [-0.56,0.30] | -0.256  [-0.70,0.14] | -0.403 *  [-0.70,-0.05] | -0.192  [-0.63,0.20] | -0.098  [-0.56,0.35] | -0.097  [-0.55,0.35] | -0.001  [-0.46,0.39] |
| SNQ_TOTAL | 0.139  [-0.31,0.48] | -0.006  [-0.47,0.32] | -0.248  [-0.61,0.13] | -0.224  [-0.61,0.16] | -0.016  [-0.46,0.33] | -0.001  [-0.40,0.39] | 0.010  [-0.43,0.44] |

Bootstrapped 95% confidence intervals for Spearman’s correlations between the characteristic path length (CPL), in the seven EEG frequency bands of interest (delta: 2–4 Hz; theta: 4–8 Hz; alpha-1: 8–10.5 Hz; alpha-2: 10.5–13 Hz; beta-1: 13–20 Hz; beta-2: 20–30 Hz; gamma: 30–40 Hz) and the neuropsychological scores (MoCA and social cognition measures). Correlation coefficients and bootstrapped 95% confidence intervals are reported. Asterisks indicate uncorrected statistical significance (*p < 0.05; **p < 0.01). Significant correlations after FDR correction are reported in bold.

MoCA= Montreal Cognitive Assessment; ESCoT= Edinburgh Social Cognition Test; ToM= Theory of Mind; COGN= Cognitive; AFF= Affective; INTER= Interpersonal norm; INTRA= Intrapersonal norm; QCAE= Questionnaire of Cognitive and Affective Empathy; SNQ= Social Norm Questionnaire; OVER= Overadhere score

**Table S4.** Bootstrapped 95% confidence intervals for Spearman’s rank correlation coefficients between cognitive scores and small-world index across EEG frequency bands.

| Small-World index (SW) | | | | | | | |
| --- | --- | --- | --- | --- | --- | --- | --- |
| Cognitive score | **Delta**  **ρ-val**  **[95% CI]** | **Theta**  **ρ-val**  **[95% CI]** | **Alpha-1**  **ρ-val**  **[95% CI]** | **Alpha-2**  **ρ-val**  **[95% CI]** | **Beta-1**  **ρ-val**  **[95% CI]** | **Beta-2**  **ρ-val**  **[95% CI]** | **Gamma**  **ρ-val**  **[95% CI]** |
| MoCA | 0.008  [-0.41,0.42] | -0.031  [-0.43,0.40] | 0.037  [-0.40,0.44] | 0.114  [-0.27,0.48] | 0.095  [-0.34,0.53] | 0.070  [-0.33,0.49] | 0.270  [-0.12,0.64] |
| ESCoT_COGN_ToM | -0.211  [-0.59,0.31] | -0.218  [-0.57,0.29] | -0.210  [-0.57,0.25] | -0.069  [-0.49,0.43] | -0.155  [-0.48,0.27] | -0.153  [-0.49,0.33] | -0.286  [-0.64,0.09] |
| ESCoT_AFF_ToM | -0.305  [-0.62,0.10] | -0.332  [-0.64,0.11] | -0.151  [-0.54,0.31] | -0.196  [-0.57,0.25] | -0.218  [-0.58,0.26] | -0.124  [-0.47,0.31] | -0.091  [-0.40,0.29] |
| ESCoT_INTER | 0.127  [-0.37,0.46] | 0.146  [-0.38,0.51] | 0.084  [-0.49,0.48] | -0.039  [-0.59,0.40] | 0.204  [-0.30,0.65] | 0.116  [-0.44,0.61] | -0.041  [-0.54,0.36] |
| ESCoT_INTRA | 0.085  [-0.41,0.52] | 0.087  [-0.34,0.52] | -0.035  [-0.41,0.43] | -0.046  [-0.43,0.41] | 0.202  [-0.17,0.55] | 0.216  [-0.14,0.54] | -0.002  [-0.39,0.42] |
| ESCoT_TOTAL | -0.175  [-0.55,0.23] | -0.183  [-0.60,0.26] | -0.101  [-0.56,0.33] | -0.126  [-0.56,0.33] | -0.014  [-0.44,0.40] | 0.019  [-0.38,0.46] | -0.174  [-0.55,0.19] |
| QCAE_COGN | -0.046  [-0.46,0.41] | 0.001  [-0.42,0.47] | -0.041  [-0.46,0.38] | -0.028  [-0.48,0.37] | 0.204  [-0.21,0.57] | 0.204  [-0.21,0.56] | 0.115  [-0.39,0.58] |
| QCAE_AFF | -0.061  [-0.51,0.41] | -0.071  [-0.52,0.42] | -0.111  [-0.53,0.34] | 0.059  [-0.34,0.50] | 0.048  [-0.36,0.50] | 0.144  [-0.28,0.57] | 0.322  [-0.07,0.65] |
| SNQ_BREAK | 0.045  [-0.39,0.50] | -0.035  [-0.44,0.46] | -0.012  [-0.40,0.52] | -0.037  [-0.44,0.56] | 0.023  [-0.40,0.51] | -0.028  [-0.45,0.47] | -0.088  [-0.47,0.44] |
| SNQ_OVER | -0.211  [-0.58,0.19] | -0.230  [-0.63,0.18] | -0.142  [-0.65,0.36] | -0.067  [-0.58,0.45] | -0.044  [-0.58,0.44] | 0.0002  [-0.50,0.51] | -0.342  [-0.68,0.16] |
| SNQ_TOTAL | -0.291  [-0.63,0.16] | -0.327  [-0.67,0.12] | -0.219  [-0.59,0.21] | -0.162  [-0.54,0.30] | -0.074  [-0.52,0.37] | -0.042  [-0.52,0.46] | -0.336  [-0.73,0.14] |

Bootstrapped 95% confidence intervals for Spearman’s correlations between the small-world index (SW), in the seven EEG frequency bands of interest (delta: 2–4 Hz; theta: 4–8 Hz; alpha-1: 8–10.5 Hz; alpha-2: 10.5–13 Hz; beta-1: 13–20 Hz; beta-2: 20–30 Hz; gamma: 30–40 Hz) and the neuropsychological scores (MoCA and social cognition measures). Correlation coefficients and bootstrapped 95% confidence intervals are reported. Asterisks indicate uncorrected statistical significance (*p < 0.05; **p < 0.01). Significant correlations after FDR correction are reported in bold.

MoCA= Montreal Cognitive Assessment; ESCoT= Edinburgh Social Cognition Test; ToM= Theory of Mind; COGN= Cognitive; AFF= Affective; INTER= Interpersonal norm; INTRA= Intrapersonal norm; QCAE= Questionnaire of Cognitive and Affective Empathy; SNQ= Social Norm Questionnaire; OVER= Overadhere score

**Table S5.** Comparison of ordinary least squares (OLS) and robust regression parameter estimates for the association between ESCoT_INTRA scores and clustering coefficient (CC) in the theta frequency band.

| PERFORMANCE METRICS | *OLS REGRESSION* | *ROBUST REGRESSION* |
| --- | --- | --- |
| root MSE | 0.876 | 0.871 |
| Regression coefficient | -30.36 | -24.19 |
| R squared | 0.442 | 0.383 |
| Adjusted R squared | 0.421 | 0.359 |

**Table S6.** Comparison of ordinary least squares (OLS) and robust regression parameter estimates for the association between ESCoT_INTRA scores and clustering coefficient (CC) in the alpha-2 frequency band.

| PERFORMANCE METRICS | *OLS REGRESSION* | *ROBUST REGRESSION* |
| --- | --- | --- |
| root MSE | 0.950 | 0.864 |
| Regression coefficient | -26.02 | -23.00 |
| R squared | 0.343 | 0.396 |
| Adjusted R squared | 0.318 | 0.372 |

**Table S7.** Comparison of ordinary least squares (OLS) and robust regression parameter estimates for the association between ESCoT_INTRA scores and characteristic path length (CPL) in the theta frequency band.

| PERFORMANCE METRICS | *OLS REGRESSION* | *ROBUST REGRESSION* |
| --- | --- | --- |
| root MSE | 0.860 | 0.830 |
| Regression coefficient | 1.47 | 1.26 |
| R squared | 0.461 | 0.437 |
| Adjusted R squared | 0.441 | 0.415 |

**Table S8.** Comparison of ordinary least squares (OLS) and robust regression parameter estimates for the association between ESCoT_INTRA scores and characteristic path length (CPL) in the alpha-1 frequency band.

| PERFORMANCE METRICS | *OLS REGRESSION* | *ROBUST REGRESSION* |
| --- | --- | --- |
| root MSE | 0.929 | 0.850 |
| Regression coefficient | 1.09 | 0.90 |
| R squared | 0.372 | 0.424 |
| Adjusted R squared | 0.348 | 0.402 |

**Table S9.** Comparison of ordinary least squares (OLS) and robust regression parameter estimates for the association between ESCoT_INTRA scores and characteristic path length (CPL) in the alpha-2 frequency band.

| PERFORMANCE METRICS | *OLS REGRESSION* | *ROBUST REGRESSION* |
| --- | --- | --- |
| root MSE | 0.916 | 0.76 |
| Regression coefficient | 1.23 | 1.08 |
| R squared | 0.389 | 0.495 |
| Adjusted R squared | 0.365 | 0.476 |

**Table S10.** Loadings of the five frequency-band estimates on all five principal components for Clustering Coefficient (CC)

| Loadings of principal components for Clustering Coefficient | | | | | |
| --- | --- | --- | --- | --- | --- |
| Frequency bands | **pca1** | **pca2** | **pca3** | **pca4** | **pca5** |
| Delta | 0.3458 | 0.7676 | -0.0404 | 0.4430 | -0.3054 |
| Theta | 0.4283 | 0.2838 | -0.3183 | -0.3680 | 0.7066 |
| Alpha-1 | 0.5100 | -0.4102 | -0.6095 | -0.0494 | 0.4447 |
| Alpha-2 | 0.4679 | -0.4018 | 0.3546 | 0.6073 | 0.3538 |
| Beta-1 | 0.4669 | 0.0218 | 0.6323 | -0.5451 | -0.2908 |

Principal component analysis (PCA) was applied to the clustering coefficient (CC) estimates obtained across the five EEG frequency bands of interest (delta: 2–4 Hz; theta: 4–8 Hz; alpha-1: 8–10.5 Hz; alpha-2: 10.5–13 Hz; beta-1: 13–20 Hz), yielding five principal components (*PC1*–*PC5*). All frequency bands showed positive loadings on *PC1*, suggesting that this component represents a broadband CC. *PC2* was mainly driven by the delta band (loading = 0.7676), indicating that it primarily captures low-frequency contributions to the CC. In contrast, *PC3* was predominantly associated with higher-frequency activity, with major contributions from alpha-1 (loading = −0.6095), alpha-2 (loading = 0.3546), and beta-1 (loading = 0.6323), thereby reflecting the influence of high-frequency oscillatory dynamics on segregation properties.

**Table S11.** Loadings of the five frequency-band estimates on all five principal components for Characteristic Path Length (CPL)

| Loadings of principal components for Characteristic Path Length | | | | | |
| --- | --- | --- | --- | --- | --- |
| Frequency bands | **pca1** | **pca2** | **pca3** | **pca4** | **pca5** |
| Delta | 0.3302 | 0.7451 | -0.0900 | 0.4377 | -0.3689 |
| Theta | 0.4181 | 0.3016 | -0.3823 | -0.3204 | 0.6967 |
| Alpha-1 | 0.5022 | -0.4442 | -0.5528 | -0.1371 | -0.4755 |
| Alpha-2 | 0.4893 | -0.3865 | 0.3091 | 0.6350 | 0.3353 |
| Beta-1 | 0.4738 | 0.0845 | 0.6668 | -0.5327 | -0.2000 |

Principal component analysis (PCA) was applied to the characteristic path length (CPL) estimates obtained across the five EEG frequency bands of interest (delta: 2–4 Hz; theta: 4–8 Hz; alpha-1: 8–10.5 Hz; alpha-2: 10.5–13 Hz; beta-1: 13–20 Hz), yielding five principal components (*PC1*–*PC5*). All frequency bands showed positive loadings on *PC1*, suggesting that this component represents a broadband CPL. *PC2* was mainly driven by the delta band (loading = 0.7451), indicating that it primarily captures low-frequency contributions to the CPL. In contrast, *PC3* was predominantly associated with higher-frequency activity, with major contributions from alpha-1 (loading = −0.5528), alpha-2 (loading = 0.3091), and beta-1 (loading = 0.6668), thereby reflecting the influence of high-frequency oscillatory dynamics on integration properties.

**Table S12.** Loadings of the five frequency-band estimates on all five principal components for Small-World Index (SW)

| Loadings of principal components for Small-World index | | | | | |
| --- | --- | --- | --- | --- | --- |
| Frequency bands | **pca1** | **pca2** | **pca3** | **pca4** | **pca5** |
| Delta | 0.4471 | 0.6950 | -0.1450 | -0.3543 | -0.4129 |
| Theta | 0.4386 | 0.2504 | -0.3378 | 0.3551 | 0.7105 |
| Alpha-1 | 0.4861 | -0.1722 | 0.2825 | 0.6766 | -0.4433 |
| Alpha-2 | 0.4778 | -0.2193 | 0.6419 | -0.4594 | 0.3171 |
| Beta-1 | 0.3783 | -0.6136 | -0.6108 | -0.2821 | -0.1666 |

Principal component analysis (PCA) was applied to the small-world index (SW) estimates obtained across the five EEG frequency bands of interest (delta: 2–4 Hz; theta: 4–8 Hz; alpha-1: 8–10.5 Hz; alpha-2: 10.5–13 Hz; beta-1: 13–20 Hz), yielding five principal components (*PC1*–*PC5*). All frequency bands showed positive loadings on *PC1*, suggesting that this component represents a broadband SW index. *PC2* was mainly driven by the delta band (loading = 0.6950), indicating that it primarily captures low-frequency contributions to the SW. In contrast, *PC3* was predominantly associated with higher-frequency activity, with major contributions from alpha-2 (loading = 0.6419), and beta-1 (loading = -0.6108), thereby reflecting the influence of high-frequency oscillatory dynamics on small-world organisation.

**Table S13.** Comparison of generalised linear models (GLM) with and without outlier detection procedures, evaluating the association between ESCoT_INTRA scores and the three principal components derived from clustering coefficient (CC) measures, while controlling for age, sex, and years of education.

| PERFORMANCE METRICS | *GLM without Cook’s distance outlier detection* | *GLM with Cook’s distance outlier detection* |
| --- | --- | --- |
| p-value GLM | 0.0052 | 0.0012 |
| root MSE | 0.986 | 1.140 |
| PC1 coefficient (p-value) | -13.61 (0.0008) | -12.44 (0.0003) |
| R squared | 0.555 | 0.672 |
| Adjusted R squared | 0.428 | 0.562 |

**Table S14.** Comparison of generalised linear models (GLM) with and without outlier detection procedures, evaluating the association between ESCoT_INTRA scores and the three principal components derived from characteristic path length (CPL) measures, while controlling for age, sex, and years of education.

| PERFORMANCE METRICS | *GLM without Cook’s distance outlier detection* | *GLM with Cook’s distance outlier detection* |
| --- | --- | --- |
| p-value GLM | 0.00354 | 0.00014 |
| root MSE | 0.966 | 1.110 |
| PC1 coefficient (p-value) | 0.6489 (0.0005) | 0.624 (0.00003) |
| R squared | 0.573 | 0.745 |
| Adjusted R-squared | 0.451 | 0.660 |

**Figure S1**

**
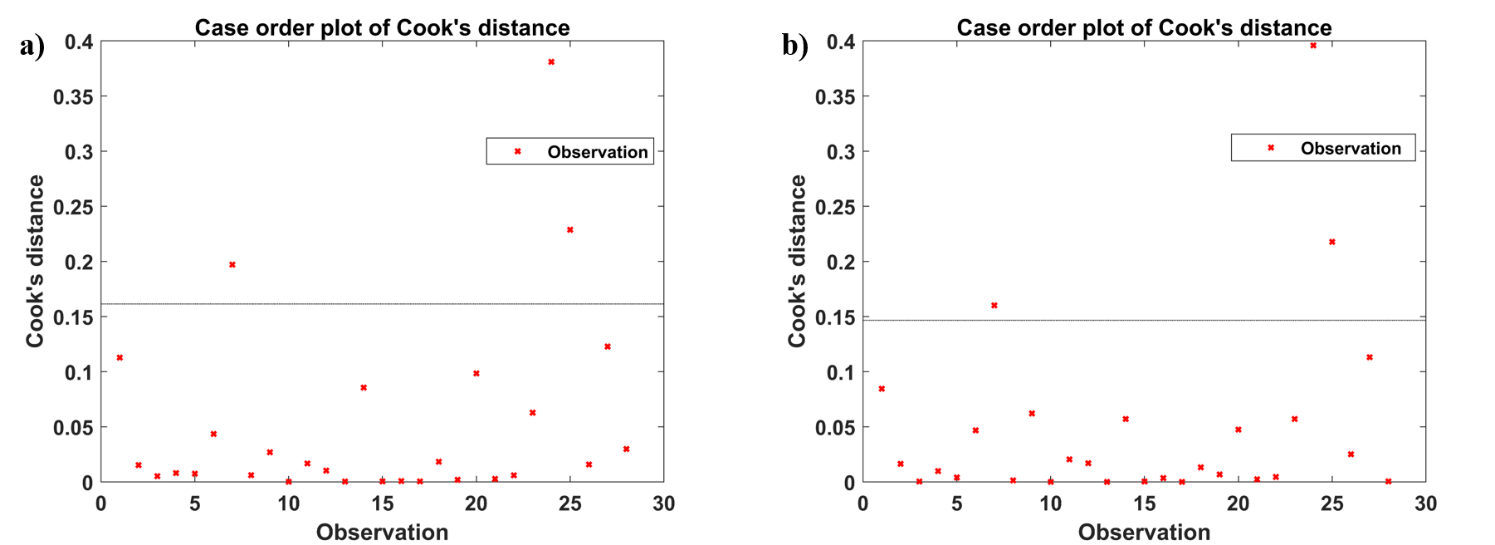
**

Cook’s distance plots for the GLM examining (a) clustering coefficient (CC) and (b) characteristic path length (CPL).

The plots display Cook’s distance values for all observations included in the generalised linear model (GLM) analyses. Observations with Cook’s distance values exceeding three times the mean Cook’s distance were classified as highly influential, indicating a potentially disproportionate impact on model parameter estimates and associated standard errors. In panel (a), corresponding to the GLM assessing the clustering coefficient (CC), three of the 28 observations exceeded this threshold: observation 7 (Cook’s distance = 0.197; deviance residual = −1.433), observation 24 (Cook’s distance = 0.381; deviance residual = −2.526), and observation 25 (Cook’s distance = 0.228; deviance residual = 1.432). In the panel (b), Cook’s distance plot for the GLM examining characteristic path length (CPL) is reported and identifies three observations as outliers in the model: observation 7 (Cook’s distance = 0.160 and deviance residual = -1.826), observation 24 (Cook’s distance = 0.395 and deviance residual = -2.460), observation 25 (Cook’s distance = 0.218 and deviance residual = 1.458).

**Figure S2**

**
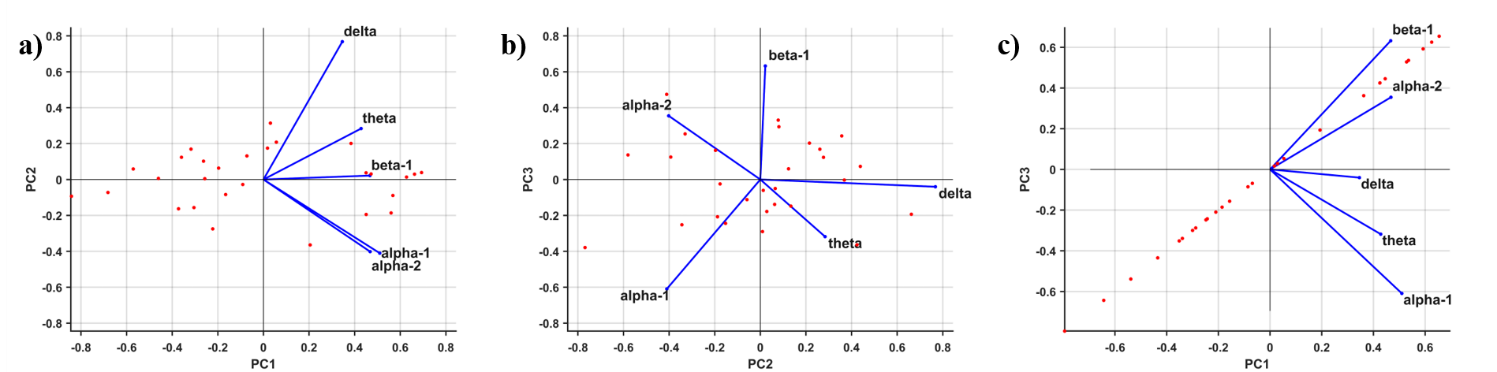
**

Principal Component Analysis (PCA) 2D Biplots for Clustering Coefficient (CC).

All biplots illustrate the loading vectors (lines) for the original estimates of CC across the five frequency bands (delta, theta, alpha-1, alpha-2, and beta-1) alongside individual observation scores (scatter points), respectively, for pc1 vs. pc2 (a), for pc2 vs. pc3 (b), for pc1 vs. pc3 (c). All EEG frequency bands contributed positively and relatively uniformly to *pc1*, supporting its interpretation as a broadband summary component, thereby reflecting the joint contribution of low-to-mid frequency oscillatory activity. The *pc2* is primarily guided by delta-band CC (loading = 0.768), reflecting the topological contribution of low-frequency rhythms. In contrast, PC3 was principally governed by high-frequency rhythms, even with opposite contributions, negative for the alpha-1 (loading = -0.610), while positive for alpha-2 (loading = 0.3546) and beta-1 (loading = 0.632) CC estimates.

**Figure S3**


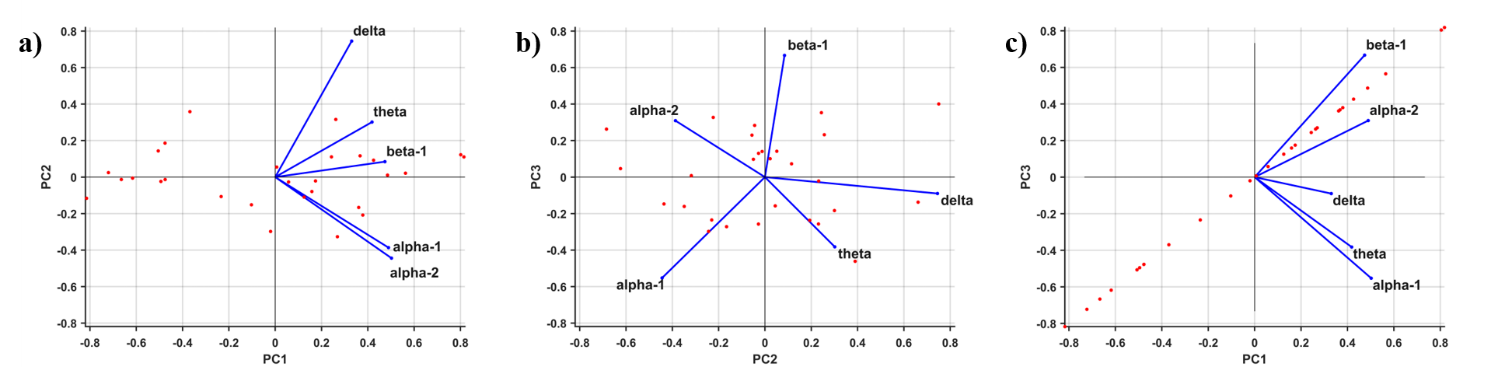


Principal Component Analysis (PCA) 2D Biplots for Characteristic Path Length (CPL).

All biplots illustrate the loading vectors (lines) for the original estimates of CPL across the five frequency bands (delta, theta, alpha-1, alpha-2, and beta-1) alongside individual observation scores (scatter points), respectively, for pc1 vs. pc2 (a), for pc2 vs. pc3 (b), and for pc1 vs. pc3 (c). All EEG frequency bands contributed positively and relatively uniformly to *pc1*, supporting its interpretation as a broadband summary component, thereby reflecting the joint contribution of low-to-mid frequency oscillatory activity. The *pc2* is primarily guided by delta-band CPL (loading = 0.7451), reflecting the topological contribution of low-frequency rhythms. In contrast, PC3 was principally governed by high-frequency rhythms, even with opposite contributions, negative for the alpha-1 (loading = -0.5528), while positive for alpha-2 (loading = 0.3091) and beta-1 (loading = 0.6668) CPL estimates.

**Figure S4**

**
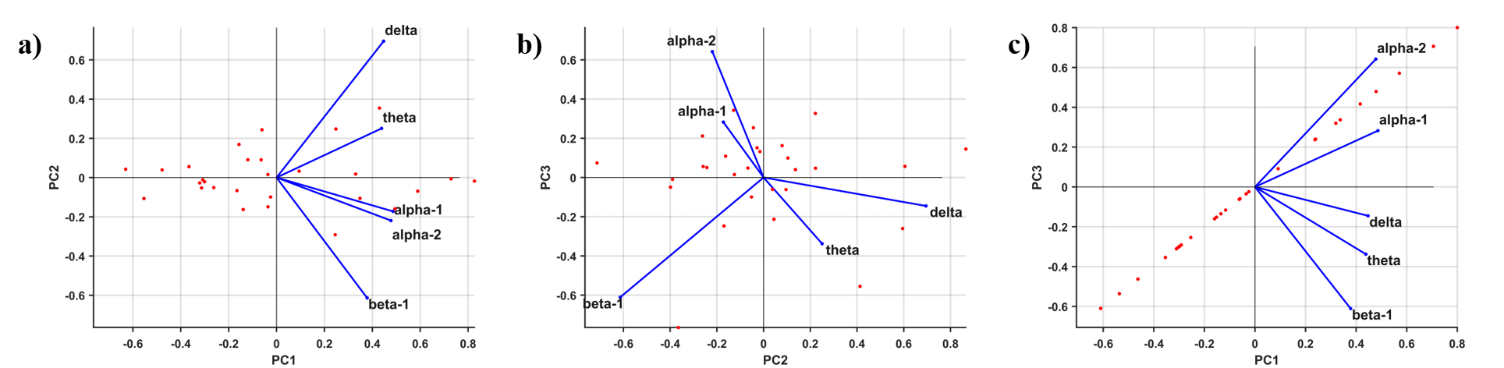
**

Principal Component Analysis (PCA) 2D Biplots for Small-World index (SW).

All biplots illustrate the loading vectors (lines) for the original estimates of SW across the five frequency bands (delta, theta, alpha-1, alpha-2, and beta-1) alongside individual observation scores (scatter points), respectively, for pc1 vs. pc2 (a), for pc2 vs. pc3 (b), for pc1 vs. pc3 (c). All EEG frequency bands contributed positively and relatively uniformly to *pc1*, supporting its interpretation as a broadband summary component, thereby reflecting the joint contribution of low-to-mid frequency oscillatory activity. The *pc2* is primarily guided by delta-band SW (loading = 0.6950), reflecting the topological contribution of low-frequency rhythms. In contrast, PC3 was principally governed by high-frequency rhythms, but with opposite directionality with respect to CC and CPL, with positive contribution for the alpha-1 (loading = 0.2825) and alpha-2 (loading = 0.6419) and negative for beta-1 (loading = -0.6108) SW estimates.
